# Development and Evaluation of Essential Oil-Based Nanoemulgel Formulation for the Treatment of Oral Bacterial Infections

**DOI:** 10.3390/gels9030252

**Published:** 2023-03-21

**Authors:** Niamat Ullah, Adnan Amin, Arshad Farid, Samy Selim, Sheikh Abdur Rashid, Muhammad Imran Aziz, Sairah Hafeez Kamran, Muzammil Ahmad Khan, Nauman Rahim Khan, Saima Mashal, Muhammad Mohtasheemul Hasan

**Affiliations:** 1 Natural Products Research Lab, Gomal Centre of Pharmaceutical Sciences, Faculty of Pharmacy, Gomal University, Dera Ismail Khan 29050, Pakistan; 2 Gomal Centre of Biochemistry and Biotechnology (GCBB), Gomal University, Dera Ismail Khan 29050, Pakistan; 3 Department of Clinical Laboratory Sciences, College of Applied Medical Sciences, Jouf University, Sakaka 72388, Saudi Arabia; 4 Nano Carriers Research Lab, Gomal Centre of Pharmaceutical Sciences, Faculty of Pharmacy, Gomal University, Dera Ismail Khan 29050, Pakistan; 5 Department of Pharmacology, Faculty of Allied Health and Pharmaceutical Sciences, Lahore College for Women University, Lahore 05422, Pakistan; 6 Department of Pharmacy, Kohat University of Science and Technology, KUST, Kohat 26000, Pakistan; 7 Department of Pharmacognosy, Faculty of Pharmacy and Pharmaceutical Sciences, University of Karachi, Karachi 75270, Pakistan

**Keywords:** essential oil, nanoemulgel, oral infection, Carbopol 940, quorum sensing

## Abstract

Prevalence of oral infections in diabetic patients is a health challenge due to persistent hyperglycemia. However, despite great concerns, limited treatment options are available. We therefore aimed to develop nanoemulsion gel (NEG) for oral bacterial infections based on essential oils. Clove and cinnamon essential oils based nanoemulgel were prepared and characterized. Various physicochemical parameters of optimized formulation including viscosity (65311 mPa·S), spreadability (36 g·cm/s), and mucoadhesive strength 42.87 N/cm^2^) were within prescribed limits. The drug contents of the NEG were 94.38 ± 1.12% (cinnamaldehyde) and 92.96 ± 2.08% (clove oil). A significant concentration of clove (73.9%) and cinnamon essential oil (71.2 %) was released from a polymer matrix of the NEG till 24 h. The ex vivo goat buccal mucosa permeation profile revealed a significant (52.7–54.2%) permeation of major constituents which occurred after 24 h. When subjected to antimicrobial testing, significant inhibition was observed for several clinical strains, namely *Staphylococcus aureus* (19 mm), *Staphylococcus epidermidis* (19 mm), and *Pseudomonas aeruginosa* (4 mm), as well as against *Bacillus chungangensis* (2 mm), whereas no inhibition was detected for *Bacillus paramycoides* and *Paenibacillus dendritiformis* when NEG was utilized. Likewise promising antifungal (*Candida albicans*) and antiquorum sensing activities were observed. It was therefore concluded that cinnamon and clove oil-based NEG formulation presented significant antibacterial-, antifungal, and antiquorum sensing activities.

## 1. Introduction

Oral microbiome is quiet diverse and is comprised of over 700 different “core” and “Transient” microbial species including Gram-positive, Gram-negative and fungal species [1], which significantly contribute towards gingivitis, periodontitis, dental caries, stomatitis, and candidiasis [2]. This greater microbial load is influenced by several factors including host, signaling systems, and environmental factors [3]. Several bacteria, fungi, viruses, or protozoa are responsible for such infections that become more severe due development of biofilms [4,5]. 

Tooth decay (dental caries) and periodontal disease are prevalent oral infections that result from bacteria accumulating on the surface of teeth. Among the most caries-causing bacterial species are *Mutans streptococci*, including *S. mutans* and *Streptococcus sobrinus*. These bacteria consume sugars in the diet and produce acid, which lowers the pH of the mouth and leads to the demineralization of tooth enamel, ultimately causing tooth decay [6].

Gingivitis is a gum infection that is the first stage of periodontal disease. It is characterized by gingival bleeding and swollen gums and mostly it is reversible. If not treated properly and swiftly, it can lead to periodontitis. Periodontitis is a chronic inflammatory illness in which the supporting components of the teeth, such as the periodontal ligament and alveolar bone, are destroyed. In the general population, severe periodontitis affects 10% to 15% of the population [7].

The complex structure of bacterial colonies in the oral cavity makes it difficult to treat bacterial infections in dentistry [8]. Oral infections are usually treated with antibiotics, antiamoebic drugs, and quaternary ammonium compounds. However, the use of these drugs may lead to the development of resistance and toxicity due to the selective pressure induced by their prolonged use [9]. Thus, there exists a great potential for alternative therapies that are safe, effective, and easy to use, such as essential oils.

The potential for using essential oils against microbial infections is gaining attention [10]. Most well-known essential oils for maintaining good dental health include lavender, eucalyptus, peppermint, clove, and cinnamon essential oils [11], which are also supported with scientific evidence [12]. Cinnamon oil has been investigated for its antibacterial action against both Gram-positive and Gram-negative pathogens. It is mainly collected from *Cinnamomum zeylanicum* (Family *Lauraceae*) [13]. The cinnamon essential oil is traditionally used in various herbal remedies due to diverse biological activities including anti-inflammatory, antidiabetic, carminative, antiviral, and antihypertensive properties [14]. Major constituents of cinnamon essential oil include cinnamaldehyde, Linalool, β-caryophyllene, eucalyptol, camphor, and cinnamyl acetate [15]. Several investigations have shown its effectiveness towards *Streptococcus mutans* and *Lactobacillus casei* (dental cavities), *Staphylococcus aureus*, *C. albicans*, *C. glabrata,* and *Enterococcus faecalis* [16]. A strong antimicrobial action of cinnamon essential oil is due to disruption of the cell membrane, which promotes leakage of intracellular components [17]. 

Clove essential oil is mainly obtained from *Syzygium aromaticum* (family *Myrtaceae*) [18] and the FDA has classified the clove essential oil safe as “generally regarded as safe” (GRAS) and this essential oil possess several properties such as antioxidant, antioxidant, antifungal, as well as antibacterial [19]. Furthermore, clove oil has also been proven to effectively inactivate bacterial strains such as *E. coli* O157:H7, *Salmonella typhimurium*, *Listeria monocytogenes*, and *S. aureus* [19].

Nanoemulsion is a drug delivery system that is designed with enhanced stability and solubility [20]. The globules size in the nanoemulsion is in the nano range and are stabilized by mixture of the surfactants and co-surfactants [21]. The globules of a nano metric size in the oil phase normally facilitate efficient drug delivery; and therefore, the formulation of nanoemulsion is promising approach against oral infections [22]. In order to improve retention in the oral cavity and even enable sustained release of a medication, nanoemulsion gels (NEGs) are prepared by adding a thickening or gelling agent (Carbopol) to a nanoemulsion [23,24]. NEGs are valuable formulations as they offer a sustained release of drugs and their mucoadhesive nature facilitates a prolonged contact time compared to nano emulsion [21,24]. NEGs, which are either W/O or O/W emulsions, are combined with a gel base to create a more stable, viscous, and non-greasy formulation [25]. This is a preferred dosage form for hydrophobic drugs. These formulations possess an improved bioavailability, as they reduce surface tension and shield incorporated drug from enzymatic degradation and hydrolysis [26]. Furthermore, an improved infusibility, greater drug loading capacity, and permeability make NEGs the most acceptable delivery system for dental drug delivery [27].

Carbopols are mainly comprised of acrylic acid polymers that are cross-linked with polyalkenyl ethers or divinyl glycol [28]. The non-irritant and non-toxic nature of carbapols make them widely acceptable for topical applications [29]. A high molecular weight limits their penetration into the skin, and they thus are considered as good substitutes for oil-based vehicles [30]. Based on the widespread prevalence of oral microbial infections and importance of NEGs, this project was designed to develop a muco-adhesive nanoemulgel for the treatment of oral bacterial infections.

## 2. Results and Discussion

### 2.1. HLB Values of Different Smix Ratios 

The HLB values of different Smix ratios (surfactant: co-surfactant) (1:1, 1:2, 1:3, 1:4, 2:1, 3:1, and 4:1) were determined and it was observed that the HLB values increased with an increase in the concentration of the surfactant whereas a significant decrease in HLB values was noticed with a decrease in the concentration of the co-surfactant (Table 1). The Smix ratio 4:1 was chosen due to its high HLB value (12.86).

It is advantageous for oil in water nanoemulsions to have higher HLB values [24]. The HBL values and particle size in o/w emulsion has inverse relationship higher the HLB value lower is the globule size of the emulsion. Furthermore, the higher HLB values are helpful for producing a uniform and fine nanoparticle [24]. As the oil concentration rises, the nanoemulsion particle size expands, and the concentration of the surfactants is forced to keep up with the oil droplets’ rising concentration [30]. (The GC-MS Profile of clove essential oil as shown in Figure S1.)

### 2.2. Optimization of Nanoemulsion

The optimization of the nanoemulsion was performed by using the pseudo ternary diagram. In this case, Smix (Span 80 and Tween 80) was combined with olive oil at the dispersed phase, and distilled water was used as the dispersing medium.

**Figure 1:**
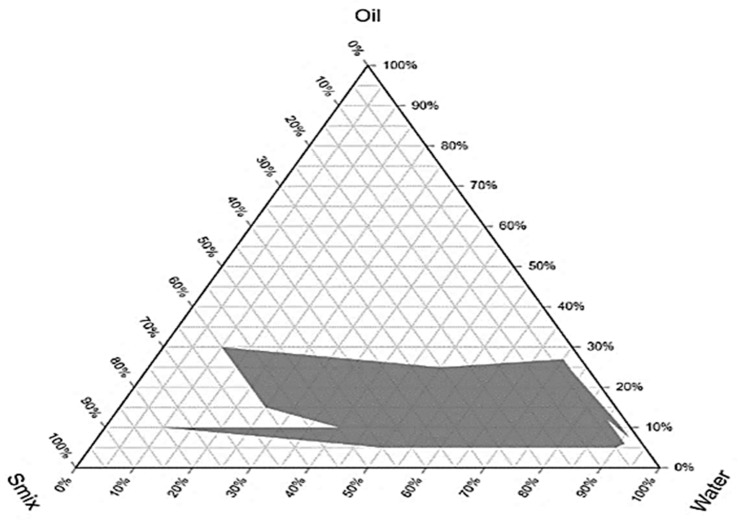
Pseudo ternary phase diagram.

The region of stable oil in water nanoemulsion is represented by the shadowed area in the built pseudo-ternary phase (Figure 1). The phase diagram’s each corner represents 100% concentration of constituent. 

### 2.3. Globule Size, Polydispersity Index, and Zeta Potential

Four formulations of the nanoemulsion were prepared with different concentrations of the oily phase. The concentration of the surfactants was also minimized for the nanoemulsion. The N1 formulation showed the least globule size of (152 nm) compared to all formulations (Table 2). Due it being the least size, N1 formulation was selected as an optimum formulation.

### 2.4. Kinetic Stability

The optimized formulation was centrifuged to check the kinetic stability. The formulation was rendered as kinetically stable since no phase separation, creaming, and flocculation occurred after centrifugation at 1000, 2000, and 3000 rpm.

### 2.5. Thermodynamic Stability

To assess the thermodynamic stability, a heating–cooling cycle was performed on the test sample. It was observed that nanoemulsion creaming and phases separating occurred after three consecutive heating–cooling cycles, which was a clear indication of thermodynamic instability. Nanoemulsions are thermodynamically unstable while being kinetically stable, and therefore creaming and phase separation occurs upon long-term storage. The centrifugation process is typically used to execute stress conditions to freshly formed nanoemulsions in order to determine their kinetic stability. This is due to fact that the colloidal dispersion’s free energy in a nanoemulsion is higher compared to the free energy of the individual phase indicates that nanoemulsion [31].

### 2.6. Optimization of Nanoemulgel

**Figure 2:**
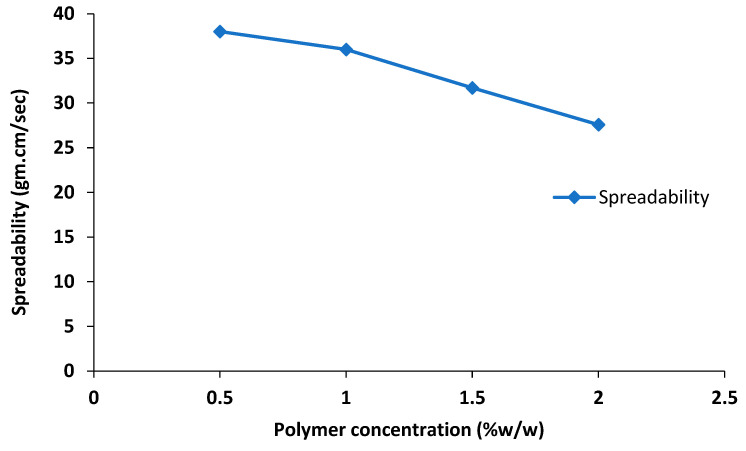
Effect of polymer concentration on spreadability of nanoemulgel.

**Figure 3:**
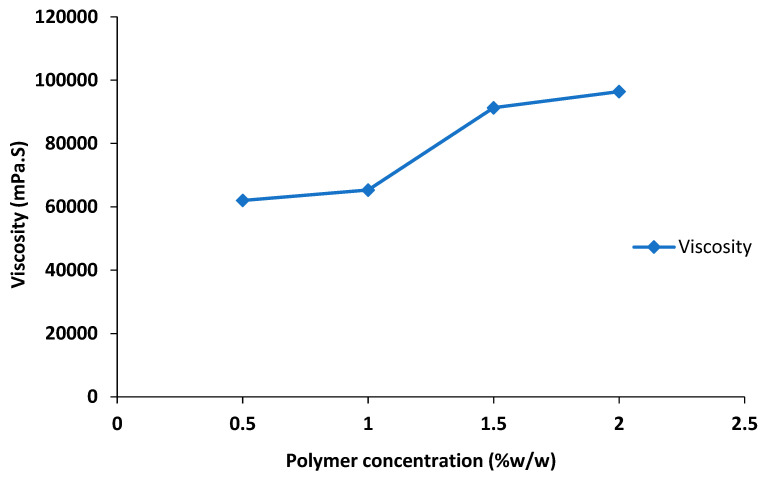
Effect of polymer concentration on viscosity of nanoemulgel.

The nanoemulgel formulations were optimized based on viscosity and spreadability and it was observed that the spreadability of the nanoemulgel decreased with an increase in the polymer concentration (Table 3, Figure 2), whereas a significant increase in viscosity of the nanoemulgel was seen with an increase in polymer concentration (Table 3, Figure 3). The formulation F1 was less viscous (62,035 ± 10 mPa·S) with a high spreadability value of 38 ± 1, whereas formulation F3 and F4 both were highly viscous, having viscosity 91,306 ± 15 and 96,432 ± 10 mPa·S, respectively, and very low spreadability. Formulation F2 was considered as optimized due to its optimal viscosity of 65,311 ± 7 mPa·S and spreadability of 36 ± 0.5.

The polymers (gelling agents) are employed to keep the nanoemulgel consistency constant. Physical homogeneity, consistency, bio-adhesive qualities, swelling index, rheological studies, drug release kinetics, extrudability, and spreadability are among the parameters that play a significant role in determining the consistency of nanoemulgel formulations, with gelling agents being a key factor in achieving these parameters [32]. High viscosity (30,000–50,000 centipoises) of synthetic polymer Carbopol 940 is typically utilized for topical or transdermal formulations as it forms a transparent gel with water or hydro alcoholic frameworks. Likewise, spreading is a crucial aspect for preparations intended for topical treatment [33]. Our findings demonstrated that the improved formulation is suitable for topical application and has acceptable spreadability properties. An increase in the concentration of the gelling agent (Carbopol 940) showed a positive effect on the viscosity of the nanoemulgel formulation; consequently, the spreadability of the nanoemulgel formulation decreased, as there is inverse relationship between the viscosity and spreadability of the formulation (Figure 2 and Figure 3) [34].

### 2.7. FTIR

**Figure 4:**
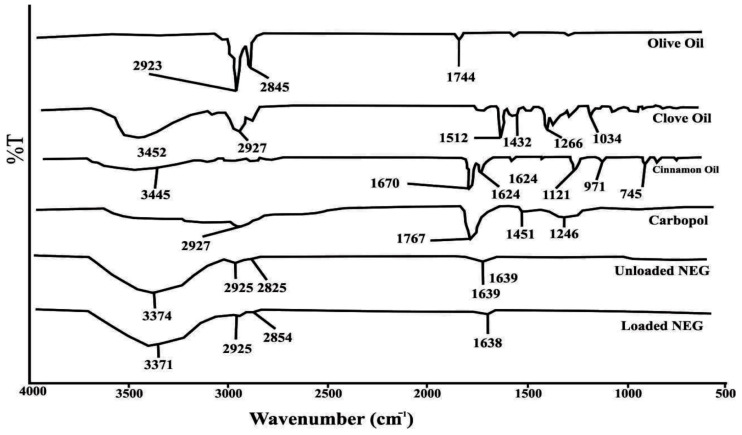
FTIR analysis of the active ingredients, excipients, blank nanoemulgel, and essential oil-loaded nanoemulgel formulation.

FTIR analysis is utilized to ascertain the drug-excipient compatibility in nanoemulgel formulations. The characteristic peaks of essential oils and other excipients, both alone and in the optimized nanoemulgel formulation, were found to match the previously reported functional groups of these constituents. For instance, in the FTIR spectrum of olive oil, peaks at 2923 cm^−1^ and 2845 cm^−1^ correspond to OH and fatty acid stretching, while a peak at 1744 cm^−1^ represents the ester C=O group (Figure 4). In clove oil, a peak at 3452 cm^−1^ represents OH stretching, and peaks at 1512 cm^−1^, 1425 cm^−1^, and 1266 cm^−1^ correspond to the aromatic C=C and phenolic group. In cinnamon oil, a peak at 3452 cm−1 represents OH stretching, a peak at 1670 cm−1 corresponds to the C=O group, a peak at 1624 cm^−1^ represents the C=C group, a peak at 745 cm−1 corresponds to the aromatic C-H group, and a peak at 970 cm^−1^ indicates C-H bending. In the FTIR spectrum of Carbopol 940 the peak at 2927 cm^−1^ represents CH2 stretching, a 1767 cm^−1^ peak is associated with COOH group, whereas 1451 cm^−1^ and 1246 cm^−1^ peaks show the presence of the acrylate back bone (Figure 4). Based on FTIR spectral data of essential oils as well as excipients, it is confirmed that the characteristic peaks of essential oils are preserved in the nanoemulgel formulations, showing the absence of any type of interaction among formulation constituents.

### 2.8. Drug Content Analysis

Drug content analysis studies revealed that essential oil contents of the optimized nanoemulgel formulation for cinnamon oil and clove oil were 94.38 ± 1.12% and 92.96 ± 2.08%, respectively.

### 2.9. Entrapment Efficiency of the Nanoemulsion and Nanoemulgel

The average entrapment efficiency of the cinnamaldehyde in the nanoemulgel was 95.78%, whereas in case of the clove oil, it was 96.45%.

Entrapment efficiency and system homogeneity are influenced by the drug’s solubility in the required oily phase, as well as by its compatibility with other ingredients. An important element that greatly effects drug encapsulation is the insolubility of the investigated chemical compounds, which can be stabilized by employing surfactants and co-surfactants. This might be due to a higher surfactant content in nanoemulsion and nanoemulgel, which can lead to smaller particle size and reduced drug entrapment [35]. Furthermore, in our case, drug partitioning improved the solubility of active ingredients from the oily phase to the aqueous phase, which reduced formulation viscosity and enhanced the diffusion phase during self-assembling. This provided a clear explanation for the formulation’s earlier record of lower active ingredient entrapment efficiency [36].

### 2.10. Drug Release Profile of Nanoemulgel

**Figure 5:**
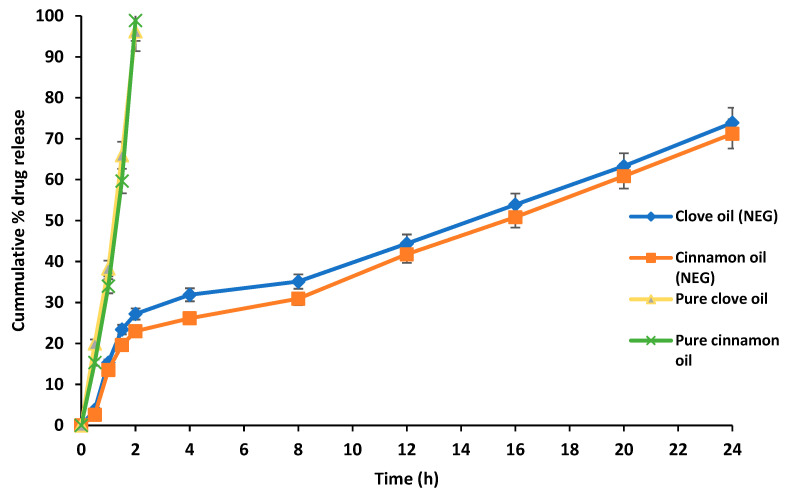
The release of cinnamaldehyde essential oil and clove essential oil from the optimized nanoemulgel formulation.

In vitro drug release profiling of nanoemulgel was performed using a Franz diffusion cell. Drug release as percentage was plotted against the experimental time (Figure 5). The essential oil-loaded nanoemulgel presented a sustained release of the drug since the essential oil was encapsulated in lipid part of the nanoemulgel. The clove essential oil-based formulation presented a significant release (73.9%) within 24 h, whereas a nearly similar release profile was recorded in case of cinnamon oil-based formulation (71.2%) (Figure 5). In the case of pure essential oils 100%, the release was observed within 2 h.

Therapeutic efficacy of any drug is dependent on the release of the drug from pharmaceutical formulation [37]. The release of the drug from the topical pharmaceutical formulation depends upon many factors such as viscosity, surfactants concentration, polymer, and drug concentration [38]. A delayed release of the essential oil from the optimized nanoemulgel formulation could possibly be due to the fact that a polymer was incorporated in the already prepared nanoemulsion. This increases the viscosity, and the structure becomes more complex. The high molecular weight of synthetic polymer Carbopol 940 results in the formation of a viscous formulation, causing a delay in the release profile of drugs from topical preparations such as nanoemulgel [32]. Moreover, it has been reported earlier that emulgel acts as a reservoir for the drug, which releases the drug initially from the internal phase to the external phase, and then into the site of the skin [39]. In nanoemulgel, the oil droplets are initially released into the gel matrix and then penetrate directly into the subcutaneous layer of the skin, bypassing the need to pass through a hydrophilic phase transfer in the nanoemulsion [40].

### 2.11. Mechanism of Drug Release from the Essential Oil-Loaded Nanoemulgel

Different kinetic models, such as Zero order, First order, Higuchi, Hixson–Crowell, and Korsmeyer–Peppas models were used to establish drug release mechanism from nanoemulgel. It was observed that our formulation followed Korsmeyer–Peppas model with an R^2^ value of 0.99. It was concluded that the drug release from the produced formulation was diffusion-controlled (Table 4).

### 2.12. Buccal Mucosa Permeation of the Prepared Nanoemulgel

**Figure 6:**
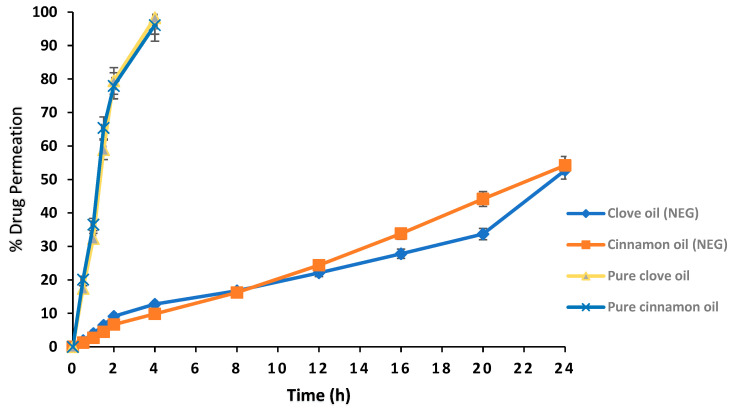
Goat buccal mucosal permeation of the essential oil from optimized nanoemulgel formulation.

A moderate permeation of cinnamon (54.21%) and clove (52.75%) essential oil was observed in the case of nanoemulsion. Overall, a delayed permeation was observed for the essential oil in nanoemulgel, which was contrary to nanoemulsion. Pure drug (100%) permeation was achieved in 4 h (Figure 6).

### 2.13. Mucoadhesive Test

Hydrophilic polymer Carbopol 940 (used as a gelling agent) also possesses significant mucoadehession strength. The mucoadhesive strength of the nanoemulgel was calculated by using a modified balance method. It was observed that the prepared nanoemulgel formulation has a very good mucoadhesive strength of 42.87 N/cm^2^.

Mucoadehession is essential to deliver the required drug concentration to the diseased mucosa as the prepared formulation stays intact to the affected area for longer time due to adhesive properties. This therefore provides a maximum time of contact to the formulation release the drug. The hydrophilic polymers possess strong mucoadehession characteristics due to the fact that they can form hydrogen bonding interaction with the mucin [41]. Mucoadehession is a key property of the hydrophilic polymers [42]. 

### 2.14. Skin Irritation Test

An essential oil-loaded nanoemulgel formulation and formalin (positive control) were applied on rats. The rats were observed for any sign of skin irritation such as skin erythema and edema formation. The Draize skin irritation scoring system was used to rate skin irritation, and as reported earlier, if score was one or less than one, then no or negligible signs of irritation were demonstrated. The skin irritation score of the nanoemulgel loaded with essential oil was <1, and there were no significant signs of skin irritation. The lack of irritancy in the tested formulation may be attributed to the fact that the essential oils were encapsulated in olive oil and the non-irritant polymer Carbopol 940 [43]. Likewise, the prepared nanoemulgel formulation was also applied on the goat buccal mucosa and no signs of mucosal irritation were observed (Table 5).

### 2.15. Biological Evaluation of Nanoemulgel

#### 2.15.1. Antimicrobial Activity

The optimized nanoemulgel formulation (loaded with 3% and 1.5% essential oil) was tested for antibacterial efficacy against isolated oral bacterial strains. The essential oil-loaded nanoemulgel formulation was found to have a very distinct zone of inhibition when used against the isolated bacterial strains (Table 6). The antibacterial activity of NEG loaded with essential oil was also checked against other bacterial strains *P. aeruginosa* (4 ± 1 mm) and *Bacillus chungangensis* (2 ± 1 mm), whereas no activity was seen against *Bacillus paramycoides* and *Paenibacillus dendritiformis*. Moreover, it was found that the antibacterial activity of the essential oil-loaded nanoemulgel against the oral bacterial strains was increased as the concentration of the EO increased from 1.5–3% *w*/*w*, and the zone of inhibition increased from 6–19 mm in both the oral bacterial strains (Supplementary Information (Figures S2 and S3). Likewise, significant inhibition of *S. aureus* and *S. epidermidis* was also recorded (Table 6). The cinnamon and clove essential oils were previously used against *E. coli* and *K. pneumoniae* with different concentrations. It was found that these essential oils were potentially active, and the zone of inhibition increased with an increase in the essential oil concentration [44]. The essential oil loaded nanoemulgel also inhibited *Candida albicans* (6 ± 1 mm). It has been reported earlier that the hydrophobic nature of the essential oils may lead to the deterioration of the membrane and increase the permeability of the membrane in bacteria [45].

#### 2.15.2. Antiquorum Sensing Activity

Antiquorum sensing of the prepared nanoemulgel was performed against *Chromobacterium voilaceum* (biomarker strain), and significant results were recorded (20 ± 1 mm) (Table 7, Supplementary Materials Figure S5).

### 2.16. Scanning Electron Microscopy of the Essential-Loaded Nanoemulgel

**Figure 7:**
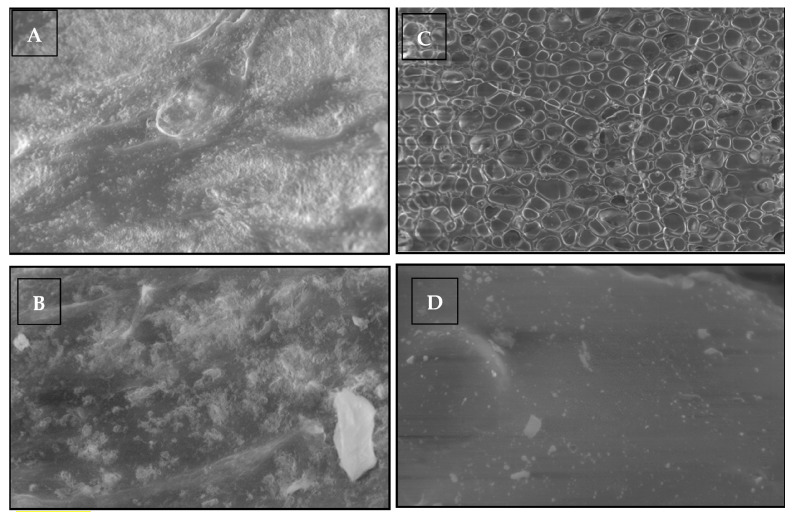
SEM of the essential oil loaded nanoemulgel (EHT = 20.00 Kv, Signal A C2DX, WD 7.5 to 9 mm, resolution (**A**) = 2.01 kx, (**B**) = 5.03 KX, (**C**) = 249 KX, (**D**) = 25 KX).

Scanning electron microscopic analysis provides the morphological information about the formulation. The SEM images confirmed that globules of the nanoemulsions are evenly distributed in the polymeric gel base of the emulgel and exhibited a well-defined spherical shape. Moreover, the porous nature of the Carbopol gel embedded the nanoemulsion globules forming a gel layer barrier that facilitates a delayed release of the drug. The spherical shape of the globules is beneficial due to their ability to squeeze easily to penetrate through the microscopic pores of the skin (Figure 7).

### 2.17. Kinetic and Thermodynamic Stability of Nanoemulgel

The optimized nanoemulgel formulation underwent characterization to determine its pH, homogeneity, and color. No significant changes were observed in the organoleptic properties of the optimized nanoemulgel formulation, and it remained homogeneous for 3 months at 25 °C as shown in Table 8. Furthermore, at temperature 8 °C and 25 °C, no change in color of the formulation was noticed (Table 8 and Table 9). Likewise, no change in the pH of the optimized formulation stored at diverse temperatures was recorded. The nanoemulgel formulation remained homogeneous at 8 °C, and a slight change in the spreadability and viscosity of the formulation was recorded. The nanoemulgel formulation was kinetically stable (Table 9) m and remained stable at 48 °C; however, the spreadability of the nanoemulgel increased and viscosity decreased followed by change in color of the formulation (from white to dark brown) (Table 10).

The viscosity of pharmaceutical formulations is a crucial factor as the release of drugs and the consistency of semisolid dosage forms rely on it [46]. We observed that viscosity of the nanoemulgel was decreased with increase in temperature from 8 °C to 48 °C. Additionally, an increase in temperature led to an increase in the spreadability of the formulation, as the viscosity decreased. This is due to the inverse relationship between viscosity and spreadability of the nanoemulgel. Viscosity of the semisolid formulation decreased with an increase in temperature [47]. Thus, room temperature (25 °C) was considered the most suitable storage of nanoemulsion gel. Increasing the temperature was found to increase the spreadability of the prepared formulation, which could be advantageous for the patient, particularly in the buccal cavity where the temperature is higher than room temperature. This improvement in spreadability can facilitate application of the formulation. As far as physical stability is concerned, the formulation stability last 3 months and no phase separation occurred after centrifugation at 1000 rpm, 2500 rpm and 3000 rpm for 15 (Table 8, Table 9 and Table 10).

## 3. Conclusions

In recent years, there has been a growing interest in developing effective methods for delivering essential oils before their incorporation into different forms of medication. In particular, the use of essential oils as potential sources of antimicrobial agents for applications in treatments, food preservation, and packaging has garnered significant attention. Commercial applications of essential oils were limited as a result of issues such as poor solubility, solvent toxicity, volatility, and a strong organoleptic taste. For the development of essential-oil-based antimicrobial nano-systems, nanoemulsion are a good option due to their biocompatibility, biodegradability, nontoxicity, and target selectivity. As a result of encapsulation and delayed release from the optimized oil in water nanoemulsion formulation, cinnamon and clove essential oil-based products demonstrated promising antibacterial and antiquorum sensing activities. Due to its mucoadhesive properties and delayed release profile, it was concluded that that converting nanoemulsion into nanoemulgel would be more advantageous due to sustained release and an enhanced time of contact due to mucoadhesive nature.

## 4. Material and Methods

### 4.1. Chemicals and Bacterial Strains

Cinnamon and clove were purchased from a local market and authenticated by a taxonomist named D.I. Khan at the Institute of Biological Sciences (IBS), Gomal University. The isolation of essential oil was achieved using Clevenger apparatus. Olive oil, Carbopol 940, and triethanolamine were purchased from Sigma Aldrich, St. Louis, MI, USA. The bacterial growth media used included Tryptic Soy Broth (Hi Media, Mumbai, India), nutrient agar (Oxoid, Hampshire, UK), and Luria-Bertani Broth (LB) (Oxoid, Hampshire, UK). The bacterial strains were isolated from dental plaques of female diabetic patients with informed consent. The ethical approval for study was obtained from the Ethical Review Board, Gomal University, Pakistan, (Approval No. 335/ERB/GU) in accordance with the WMA declaration of Helsinki and ARRIVE guidelines for animals. The bacterial strains were isolated from dental plaques of female diabetic patients with informed consent and standard microbiological methods were used to isolate and purify bacteria. The Congo red agar method was employed to verify the existence of biofilm production. Genomic DNA extraction from bacteria, agarose gel electrophoresis, and PCR was performed followed by 16S rRNA gene sequencing, which was performed in the National Culture Collection of Pakistan (NCCP). The genes alignment was performed for an exact match with the NCBI nucleotide database using nBLAST. The strains were identified as *Staphylococcus epidermidis*, *Staphylococcus aureus*, *Pseudomonas aeruginosa*, *Bacillus chungangensis*, *Bacillus paramycoides* and *Paenibacillus dendritiformis* (Specimen deposited in Pakistan culture bank). The *Chromobacterium violaceum* (DSM 30191) was purchased from DSMZ, Braunschweig, Germany.

### 4.2. Essential Oil Extraction

Essential oils (cinnamon and clove) were extracted in lab using a hydrodistillation method. Detailed component analysis information was recorded (Supplementary Materials).

### 4.3. Determination of HLB values

The Hydrophilic Lipophilic Balance (HLB) was employed to calculate the amount of surfactants required for an oil to remain in a solution. The HLB value indicates the optimal ratio of surfactants necessary to form a stable emulsion, whether it is an oil in water (o/w) or water in oil (w/o) type of emulsion. The HLB required for a stable emulsion can be calculated using Equation [48], as shown in Table 11.

(1) $$HLB=\left(Wa\times HLBa\right)+(Wb\times HLBb)/Wa+Wb$$

where, Wa represents the amount (weight) of the first emulsifier, Wb shows amount (weight) of the 2nd emulsifier, and HLBa and HLBb are the representing the hydrophilic lipophilic balance of emulsifier a and b, respectively.

### 4.4. Pseudo Ternary Phase Diagram

Using a standard protocol [49], a pseudo ternary phase diagram was constructed to analyze the phase behavior of the several formulation components. In a short, 100 mL of a 4:1 surfactant (Tween 80) and co-surfactant (Span 80) mixture (Smix) were prepared, and 16 various combinations of oil (olive oil) and Smix were made in unique volume ratios ranging from 1:9 to 9:1 (1:9, 1:8, 1:7, 1:6, 1:5, 1:4, 1:3, 1:2, 1:1, 2:1, 3:1, 4:1, 5:1, 6:1, 7:1, 8:1, 9:1). The mixture of the Smix and olive oil was titrated against distilled water. Visual inspection was performed after each 5% addition of aqueous phase to the oil and Smix mixture, and the results were recorded. Finally, data were recorded after the fractions of water, oil, and Smix were selected and designed on a phase diagram with one axis characterizing the aqueous phase, the other representing the oil phase, and the third representing the Smix. On the basis of succeeding points, distinct formulations were selected from each phase diagram produced for various Smix ratios. The oily phase was selected in a such concentration to easily dissolve the 3% essential oil which was used as the active ingredient. The oil concentration from the phase diagram was chosen as the numeral of five (5%, 10%, 15% and 20%) and lowest concentration of surfactant was taken for nanoemulsion preparation.

### 4.5. Preparation of Nanoemulsion

The preparation of essential oil-loaded nanoemulsions involved blending 3% w/w of the essential oil with 5%, 10%, 15%, and 20% of olive oil using a vortex mixer and the appropriate Smix ratios. Water was then added and mixed until a stable emulsion was obtained [50].

### 4.6. Globules Size

Zetasizer 1000 HS (Malvern instrument, Worcestershire, UK) was used to determine the globule size of the prepared nanoemulsion formulation [51]. The light scattering was seen at 25 °C at a scattering angle of 90°.

### 4.7. Stability of Nanoemulsion

#### 4.7.1. Kinetic Stability

The kinetic stability of formulation was determined using centrifugation method [52]. The centrifugation of samples was performed at 1000, 2000, and 3000 rpm for 15 min at room temperature (25 °C). The appearance of the emulsion and phase separation before and after the centrifugation cycle was recorded to measure the formulation’s kinetic stability.

#### 4.7.2. Thermodynamic Stability

Thermodynamic stability of the nanoemulsion was determined by using cooling-heating cycle [53]. Briefly samples were held at 4 °C for 48 h and then transferred to 48 °C for 48 h. The nanoemulsion was then examined for any changes, such as phase separation.

### 4.8. Preparation of Nanoemulgel

Following major steps were used during preparation of nanoemulgel [29].

#### 4.8.1. Preparation of the Gel

To create the gel base, varying concentrations (0.5%, 1%, 1.5%, and 2%) of the gelling agent Carbopol 940 were mixed with distilled water and continuously stirred until the gelling agent fully dissolved at room temperature (25 °C). The use of different concentrations of the gelling agent was explored to investigate their influence on the viscosity and spreadability.

#### 4.8.2. Incorporation of the Prepared Gel into Already Prepared Nanoemulsion

Prepared nanoemulsion was added dropwise into the prepared gel and stirred for approximately 20 min at room temperature (25 °C) to convert it into emulgel. Finally, the pH of the emulgel was adjusted as the pH of the buccal cavity.

### 4.9. Optimization of the Nanoemulgel Formulation

Nanoemulgel formulation was optimized based on viscosity, spreadability and concentration of gelling agent. In order to optimize the formulation, four different formulations were prepared with varying concentration of gelling agent (0.5, 1, 1.5 and 2%) (Table 12).

### 4.10. Characterization of Nanoemulgel

#### 4.10.1. FTIR Analysis

FTIR analysis was performed with aim to see any incompatibility between the excipients and active components of the formulation as described earlier [30]. The FTIR analysis of olive oil, cinnamon essential oil and Carbopol 940 performed in both loaded and unloaded formulations of nanoemulgel.

#### 4.10.2. Viscosity

The viscosity of all formulations was determined using standard procedure [53]. The Brookfield viscometer spindle no. 4 was used to determine the viscosity of nanoemulgel at 25 ± 2 °C and 6 rpm (Digital, Labtronics, Panchkula, India).

#### 4.10.3. Physical Appearance

All the prepared nanoemulgel formulations were observed visually for their color, homogeneity, consistency, and phase separation [54]

#### 4.10.4. pH Determination

The pH of nanoemulgel formulations was determined using previously described method [54]. The pH was determined by using pH-meter by putting the tip of the electrode into the emulgel without dilution till 2 min and the result was recorded.

#### 4.10.5. Spreadability

A modified method was adopted to determine the spreadability of the formulations [55]. Briefly, two glass slides (7.5 cm length and 2.5 cm width) were employed, one was fixed to the wooden board and the other was movable, with a thread running through a pulley and holding a weight. Afterwards, the formulation was sandwiched between the two plates. A weight of 100 g was placed on the upper slide for 1–2 min to release trapped air between the slides and create a homogeneous layer of the formulation. Later, the weight (100 g) was removed, and a 30 g weight was placed over the pulley to apply a pull to the top slide. The results were recorded in seconds and expressed as the time it took a moving slide to move a predetermined distance (6.5 cm). Spreadability was calculated using the following formula:

(2) S=M×L/T

where M is the weight attached to the upper slide, L is the length of the glass slides, and T is the time it takes to separate the slides.

#### 4.10.6. Scanning Electron Microscopy of Nanoemulgel

The size and surface morphology of the nanoemulgel inner oil phase was determined using field emission scanning electron microscopy (FE-SEM LEO 1525 ZEISS) with electron high tensions of 5 and 15 kV. To capture the images, the material was placed on stubs with double-sided carbon tape and coated with an 8 nm layer of chromium. The SEM images were taken at magnifications of 1.00 KX using secondary electron (SE) and in-lens detectors. The Image J software was utilized to calculate the average size distribution of droplets based on the SEM images. [56].

#### 4.10.7. Muco-Adhesion Test

Mucoadhesive properties of nanoemulgel were determined according to a previously described method [57]. Briefly, goat buccal mucosa was obtained from the local slaughterhouse, placed in phosphate buffer (pH 6.8), and immediately transferred to the lab. After that, one pan of the balance was removed, and a vial was attached so one piece of the buccal mucosa was attached on the vial, whereas the other piece of the buccal mucosa was attached to another vial fixed on the table surface. The nanoemulgel was sandwiched between the two vials and pressed to remove any air in between the vials. Afterwards, a weight was added to the other pan of the balance until the two vials became detached from one another. The weight, on which detachment of the vials occurred, was recorded, and the muco-adhesive strength of the nanoemulgel was determined by the following formula [58].

(3) $$Mucoadhesive\;strength=\frac{weight\;\left(grams\right)\times Gravitaional\;acceleration\;(\frac{m}{s^{2}})}{Area\;of\;the\;vial\;opening\;({cm}^{2})}$$

Weight was load applied on pan to detach the pan; gravitational acceleration is 9.8 m/s^2^.

#### 4.10.8. Drug Contents Analysis

A spectrophotometric approach (UV-Vis) [55] was used to calculate the essential oil contents. After being diluted with ethanol (1:1000), homogenized in an ultrasonic bath, and measured at 230 nm for clove essential oil (eugenol) [59] and 290 nm for cinnamon essential oil, nanoemulgel formulations were used (cinnamaldehyde) [60]. From the standard curve, the essential oil content was calculated.

#### 4.10.9. Encapsulation Efficiency of Nanoemulsion

The effectiveness of the nanoemulgel encapsulation was assessed using the previously reported approach with a minor modification [61]. It was calculated by comparing the quantifiable free essential oil to the original amount of the oil that was added to the formulation. The encapsulation efficiency was calculated by using the following:

(4) EE(%)=[(initial conc. of EO−free EO)/initial conc. of EO]×100

where EE = encapsulation efficiency, EO = essential oil.

#### 4.10.10. Drug Release Profile

Release profile was determined using a Franz diffusion cell (Permegear, model 4G01-00-05-05) [62] equipped with a 7 mL acceptor compartment capacity and a diffusion area of 0.2 cm^2^ was used for the release investigation. Initially, cellulose membranes (14 kDa, dialysis tubing cellulose membrane (Sigma-Aldrich, St. Louis, MI, USA) were soaked in receptor fluid for 24 h at 37 ± 1 °C, followed by placement in donor and receptor compartments. To mimic physiological conditions, a buffer phosphate solution (pH 6.8) was used, and samples were obtained at regular intervals (0.5, 1, 1.5, 2, 4, 8, 12, 16 and 24 h). Collected samples were analyzed for cinnamaldehyde using a UV spectroscope set to 230 nm for eugenol [59] and 290 nm for cinnamaldehyde [60]. The cumulative amount of essential oil released vs. time was plotted to quantify clove oil and cinnamon oil release across the membrane. A similar procedure was repeated with pure essential oils.

#### 4.10.11. Ex vivo Permeation

An ex vivo permeation study was performed by using the goat buccal mucosa [63]. The buccal mucosa was obtained from goat and placed in a phosphate buffer pH 6.8. The buccal mucosal membrane was separated from the underlying tissues through sharp surgical blades by a registered veterinarian doctor. The buccal membrane was placed between the donor and acceptor compartments of the Franz diffusion cell. A total of 0.5 g of the emulgel was placed in the donor compartment and the Franz diffusion cell was maintained at 37 ± 1 °C. The acceptor compartment was filled with phosphate buffer pH 6.8, and sampling was performed at different time intervals (0.5, 1, 1.5, 2, 4, 8, 12, 16 and 24 h). The permeated essential oil concentration was analyzed by using spectrophotometer at 290 nm for cinnamon and 230 nm for clove oil.

#### 4.10.12. Mucosal and Skin Irritation Test

A skin irritation test was performed using previously described protocol [64]. Briefly, Wister rats (200–250 g) were divided into three groups. A 0.8% *v/v* aqueous solution of formalin was used as the positive control, whereas blank nanoemulgel formulation was used as negative control. Group 1 was the positive control (formalin group), group 2 was negative control (blank nanoemulgel formulation group), whereas group 3 was the test group (the essential oil-loaded nanoemulgel formulation group). Wister rats were subjected to a skin irritation test using the Draize scoring method (Table 13) [65,66]. Furthermore, the essential oil-loaded nanoemulgel formulation was applied to goat buccal mucosa to check the mucosal irritation. Animals were checked for evidence of erythema and edema after the standard irritant and test formulations were removed, and responses were graded at 0 h, 24 h, and 48 h. The above-mentioned test was performed to check any sort of irritation on the skin in general if the same formulation would apply to skin and soft tissue infections.

#### 4.10.13. Stability Studies

Stability of the semisolid the nanoemulgel preparation was determined [67] for 3 months and the optimized formulation was kept at different temperatures: 8 °C, 48 °C + 75% relative humidity (RH), and at room temperature (RT). Samples were characterized for organoleptic changes, physical stability, viscosity, and spreadability after each 15th day.

### 4.11. Biological Evaluation of Nanoemulgel

#### 4.11.1. Antimicrobial Property

A modified disc diffusion method [68] was used to determine the diameter of the zone of inhibition on petri plates (90 mm) using Mueller–Hinton and Sabouraud agar media. After dying for some time, the 24 h old strain of the bacteria fungal strain was spread on the media. After that, filter paper discs with a diameter of 6 mm were cut and sterilized before being carefully dropped in the center. Afterwards, 12 µL of essential oil for antibacterial testing was put on these discs and allowed to stay for 30 min. Then, the plates were incubated in an oven at 37 °C for 24 h to 48 h. Afterwards, the inhibition diameters were measured around the disc, and results were recorded.

#### 4.11.2. Antiquorum Sensing Activity

A standard method [69] was used to assess the ability of isolated drugs to disrupt quorum sensing. In petri dishes, 24 h old strain of the *C. violaceum* (1/100 ratio) was streaked onto the LB agar. After that, 6 mm filter paper discs were placed in the center of the petri dishes which were already streaked with *C. violaceum*. Afterwards, 15 microliters of the test sample was applied on the filter paper discs and were kept drying for half an hour. Finally, the plates were placed in the incubator to incubate for 24 h at 30 °C. After 24 h, the zone of inhibition was measured, and results were recorded.

### 4.12. Data Analysis

The mean ± SD was used to produce all of the data. The statistical analyses were performed by using One-Way ANOVA by using Microsoft EXCEL.

## Figures and Tables

**Table 1:** Effect of Smix ratio on HLB value.

Smix Ratio	HLB Values
1:1	9.65
1:2	7.83
1:3	6.975
1:4	6.44
2:1	11.76
3:1	12.33
4:1	12.86

**Table 2:** Particle size, zeta potential, and polydispersity index of the four formulations of nanoemulsion.

F/Code	Composition (% *w*/*w*)	P.S (nm)	Z.P (mV)	PDI
N1	Oil = 10% Smix = 2.5% *w*/*w* water = 87.5% *w*/*w*	152 ± 3	−19.54 ± 2	0.309 ± 0.023
N2	Oil = 15% *w*/*w* Smix = 3% *w*/*w* Water = 82% *w*/*w*	227 ± 2	−17 ± 3	0.267 ± 0.032
N3	Oil = 20% *w*/*w* Smix = 3.5% *w*/*w* Water = 76.5	257.6 ± 4	−14 ± 2	0.279 ± 0.055
N4	Oil = 25% *w*/*w* Smix = 3.5% *w*/*w* Water = 71.5% *w*/*w*	325 ± 3	−12.75 ± 2	0.076 ± 0.098

P.S = particle size, Z.P = Zeta potential, PDI = Polydispersity index.

**Table 3:** Physicochemical properties of nanoemulgel.

Parameters	F1	F2	F3	F4
Color	White	White	White	White
Consistency	Good	Good	Good	Good
Homogeneity	Excellent	Excellent	Excellent	Excellent
pH	6.8 ± 1	6.8 ± 0.5	6.8 ± 0.5	6.8 ± 0.5
Viscosity (mPa·S)	62,035 ± 10	65,311 ± 7	91,306 ± 15	96,432 ± 10
Spreadability (g·cm/s)	38 ± 1	36 ± 0.5	31 ± 2	27 ± 2

**Table 4:** Kinetic models used for mechanistic studies of nanoemulgel.

Formulation	Active	Zero Order	First Order	Higuchi Model	Korsmeyer–Peppas Model	N	Best Fit Model
		R2	R2	R2	R2		
Nanoemulgel	Cinn	0.9539	0.9641	0.9709	0.9904	0.507	Korsmeyer–Peppas models
	Eug	0.9515	0.9719	0.9701	0.9905	0.522	

Cinn = cnnamaldehyde, Eug = eugenol.

**Table 5:** Goat buccal mucosal and skin irritation results.

Formulation	Erythema			Edema		
	0 h	24 h	48 h	0 h	24 h	48 h
Positive control	0.00	3.7	2.80	0.00	1.69	2.13
NEG	0.00	0.30	0.00	0.00	0.00	0.00
Negative control	0.00	0.00	0.00	0.00	0.00	0.00

NEG = Nanoemulgel, positive control = formalin, negative control = blank formulation.

**Table 6:** Antimicrobial properties of essential oil loaded nanoemulgel.

F/Code	Concentration (%)	Strain	Zone of Inhibition (mm)
^1^ ENEG	3	*S. epidermidis*	19 ± 1
ENEG	3	*S. aureus*	19 ± 0.5
ENEG	1.5	*S. epidermidis*	6 ± 1
ENEG	1.5	*S. aureus*	6 ± 1
ENEG	3	*Pseudomonas aeruginosa*	4 ± 1
ENEG	3	*Bacillus chungangensis*	0
ENEG	3	*Bacillus paramycoides*	0
ENEG	3	*Bacillus chungangensis*	2 ± 1
ENEG	3	*Paenibacillus dendritiformis*	0
ENEG	3	*Candida albicans*	6 ± 1

^1^ Essential oil nanoemulgel.

**Table 7:** Antiquorum sensing activity of essential oil loaded emulsion and emulgel.

Formulation	Strain	Zone of Inhibition (mm)
^1^ ENEG	^3^ CV	20 ± 1
^2^ NEG	CV	0

^1^ Essential oil nanoemulgel, ^2^ Nanoemulgel, ^3^ *Chromobacterium violaceum*.

**Table 8:** Stability of nanoemulgel at room temperature (25 °C).

Weak	Homogeneity	pH	Spreadability	Viscosity (mPa·S)	Centrifugation	Color
0	Homogeneous	6.8 ± 0.1	37 ± 0.5	65311 ± 5	Stable	White
3rd	Homogeneous	6.8 ± 0.1	37 ± 0.3	65612 ± 8	Stable	White
6th	Homogeneous	6.8 ± 0.1	37 ± 0.5	65730 ± 10	Stable	White
9th	Homogeneous	6.8 ± 0.1	37 ± 0.1	65921 ± 5	Stable	White
12th	Homogeneous	6.8 ± 0.1	37 ± 0.1	65992 ± 8	Stable	White

**Table 9:** Stability of nanoemulgel at 8 °C.

Weak	Homogeneity	pH	Spreadability	Viscosity (mPa·S)	Centrifugation	Color
0	Homogeneous	6.8 ± 0.1	37 ± 0.5	65,311 ± 10	Stable	White
3rd	Homogeneous	6.8 ± 0.1	37 ± 0.1	66,943 ± 6	Stable	White
6th	Homogeneous	6.8 ± 0.1	36 ± 0.2	69,211 ± 7	Stable	White
9th	Homogeneous	6.8 ± 0.1	33 ± 0.3	71,388 ± 9	Stable	White
12th	Homogeneous	6.8 ± 0.1	30 ± 0.5	75,218 ± 5	Stable	White

**Table 10:** Stability of nanoemulgel at 48 °C.

Weak	Homogeneity	pH	Spreadability	Viscosity (mPa·S)	Centrifugation	Color
0	Homogeneous	6.8 ± 0.1	37 ± 0.5	65,311 ± 11	Stable	White
3rd	Homogeneous	6.8 ± 0.1	37 ± 0.8	62,230 ± 8	Stable	Off white
6th	Heterogeneous	6.8 ± 0.1	38 ± 1	58,963 ± 9	Unstable	Dark brown
9th	Heterogeneous	6.8 ± 0.1	40 ± 0.5	55,871 ± 5	Unstable	Dark brown
12th	Heterogeneous	6.8 ± 0.1	41 ± 0.9	48,222 ± 9	Unstable	Dark brown

**Table 11:** Application of surfactants depends upon the HLB value.

HLB	Application
4–6	w/o emulsifier
7–9	Wetting agents
8–18	o/w emulsifier
13–15	Detergent
10–18	Solubilizes

**Table 12:** Composition of the four formulations of nanoemulgel.

Ingredients (% *w*/*w*)	F1	F2	F3	F4
Essential oil (% *w*/*w*)	1.5	1.5	1.5	1.5
Carbopol 940 (% *w*/*w*)	0.5	1	1.5	2
Methyl paraben	0.01	0.01	0.01	0.01
Propyl paraben	0.05	0.05	0.05	0.05
Triethanolamine	q.s.	q.s.	q.s.	q.s.
Distilled water	q.s.	q.s.	q.s.	q.s.

**Table 13:** Draize scoring for skin irritation.

Evaluation of Dermal Reaction			
Value	Erythema	Value	Edema Formation
0	No erythema	0	No edema
1	Very slight erythema	1	Very slight edema
2	Slight erythema	2	Slight edema
3	Moderate to severe erythema	3	Moderate to severe edema
4	Severe erythema	4	Severe edema

## Data Availability

Not applicable.

## Supplementary Materials

The following supporting information can be downloaded at: https://www.mdpi.com/article/10.3390/gels9030252/s1, Figure S1: GC-MS Profile of clove essential oil (A) and zoom view of smaller peaks (B) from RT 13.53-16.91 [69]; Figure S2: (A,B) activity of 3% essential oil loaded nanoemulgel against *S. epidermidis* and *S. aureus* respectively; Figure S3: (A,B) activity of 1.5% essential oil loaded nanoemulgel against *S. epidermidis* and *S.aureus* respectively; Figure S4: (A,B) is activity of nanoemulgel against *Pseudomonas aeruginosa* and *Bacillus chungangensis* respectively; Figure S5: (A,B) Antiquorum sensing activity of essential oil loaded and un loaded nanoemulgel respectively against *Chromobacterium voilaceum*; Table S1: GC-MS analysis of Clove essential oil [69]; Table S2: GCMS component analysis of Cinnamon essential oil [17].
