# Supplementary material for: Development and Evaluation of Essential Oil-Based Nanoemulgel Formulation for the Treatment of Oral Bacterial Infections
Source: Gels. 2023 Mar 21;9(3):252. doi: 10.3390/gels9030252 (PMC10048686; doi:10.3390/gels9030252)
Supplement: Supplementary file 1 [file gels-09-00252-s001.zip › gels-2261329-supplementary.pdf]

gels

MDPI

Supplementary data

1. GCMS Analysis of Clove essential Oil

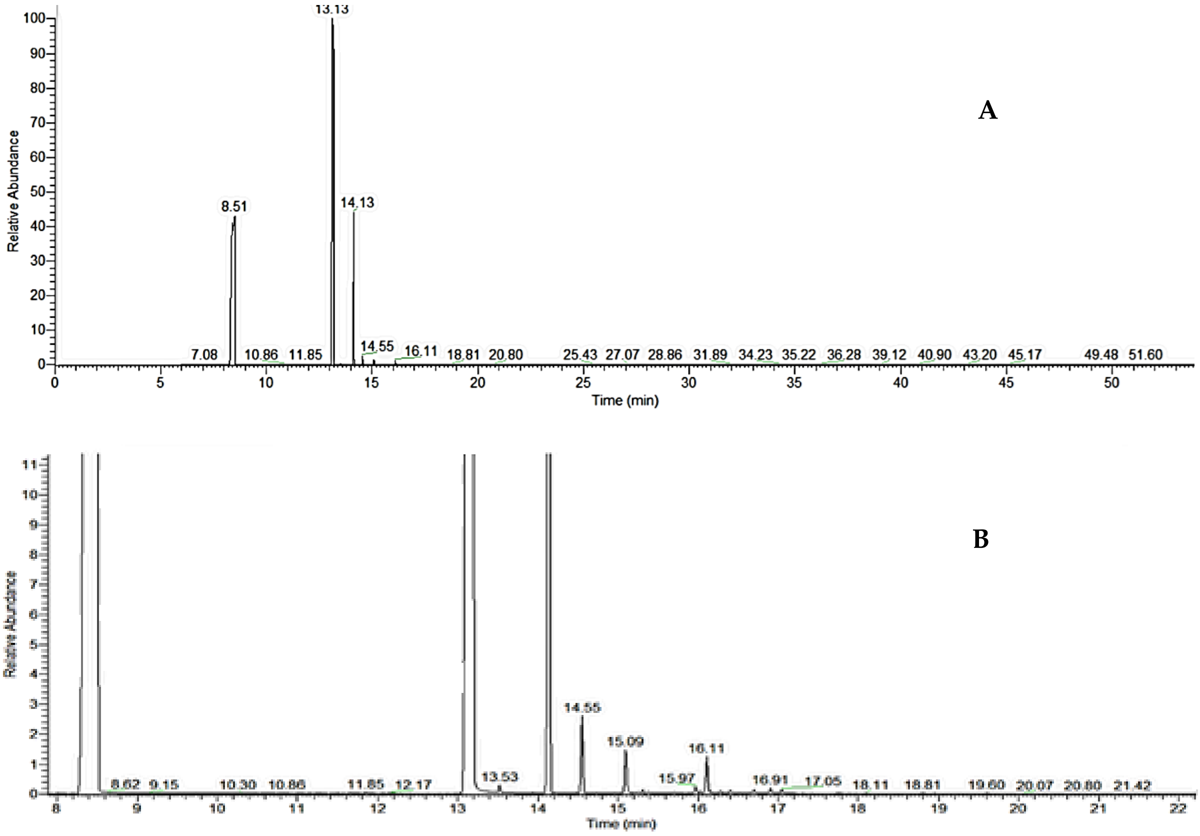

Figure S1. GC-MS Profile of clove essential oil (A) and zoom view of smaller peaks (B) from RT 13.53-16.91 [69].

Table S1. GC-MS analysis of Clove essential oil. [69]

S.No	Compound	Retention time	RI	Conc %
1	Benzyl alcohol	8.50	1025	traces
2	Methyl salicylate	10.86	1021	0.05
3	Limonene	11.66	1021	traces
4	Cubebene	11.86	1022	traces
5	Eugenol	13.13	1132	75
6	Iso-eugenol	13.53	1011	11.08
7	Caryophyllene	14.13	1322	10.2
8	α-Humulene	14.55	1422	1.22
9	Eugenol acetate	15.09	1357	12.2
10	2-carene	16.11	traces	traces
11	β-Humulene	16.28	1021	0.84
12	Cubenol	16.40	1024	0.02
13	α-Farnesene	16.70	1254	0.21
14	Caryophylene oxide	16.91	1233	0.34

Table S2. GCMS component analysis of Cinnamon essential oil [17].

S.No	Compound	Retention time	RI	Conc %
1	α-Pinene	5.66	899	1.3
2	Benzaldehyde	6.40	963	0.3
3	p-Cymene	7.82	1025	1.9
4	Limonene	7.93	1075	1.2
5	Eucalyptol	8.08	1084	5.4
6	c-Terpinene	8.66	1121	0.4
7	Linalool	9.86	1188	7
8	Isoborneol	11.64	1275	0.8
9	(E)-cinnamaldehyde	15.22	1414	71.5
10	Eugenol	16.90	1469	4.6
11	β-Caryophyllene	18.58	1518	6.4
12	Acetic acid, cinnamyl ester	19.23	1536	0.5
13	α-Humulene	19.47	1543	1.7
14	δ-Cadinene	20.97	1581	1.4
15	trans-Calamenene	21.10	1585	0.7
16	Caryophyllene oxide	22.61	1621	0.5
17	Benzyl benzoate	26.82	1710	0.5

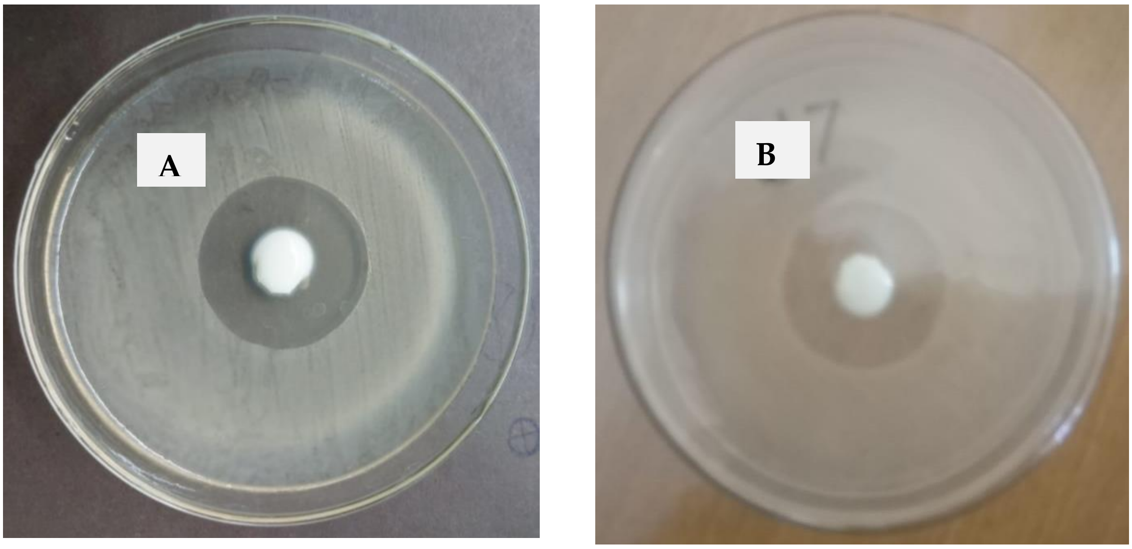

Figure S2. (A and B) activity of 3% essential oil loaded nanoemulgel against *S. epidermidis* and *S. aureus* respectively**.**

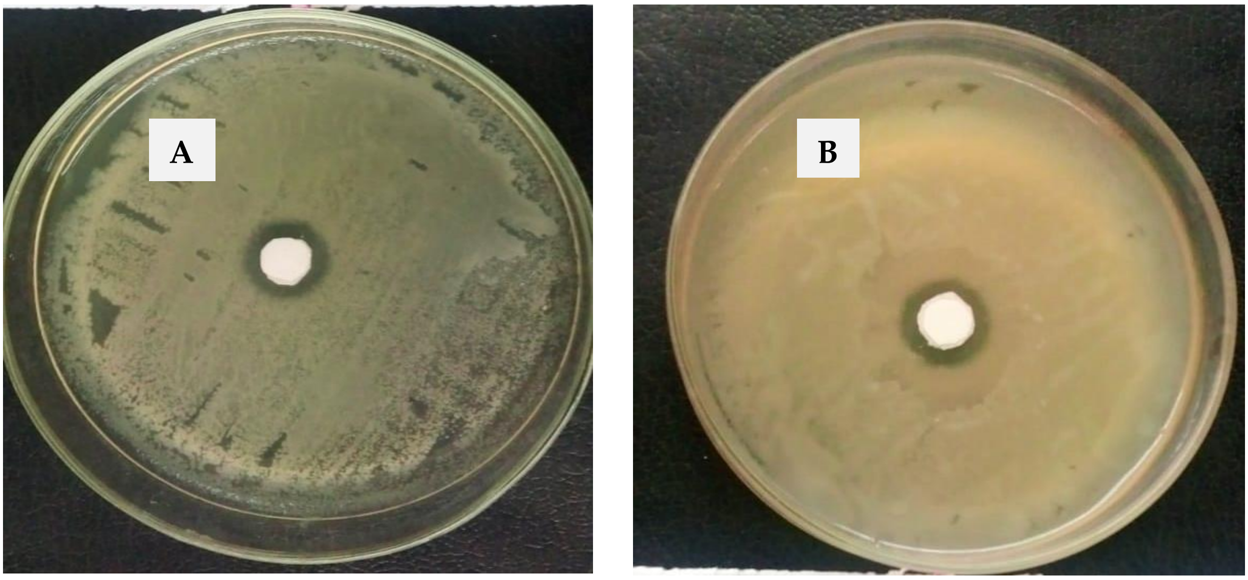

Figure S3. (A and B) activity of 1.5% essential oil loaded nanoemulgel against *S.epidermidis* and *S.aureus* respectively.

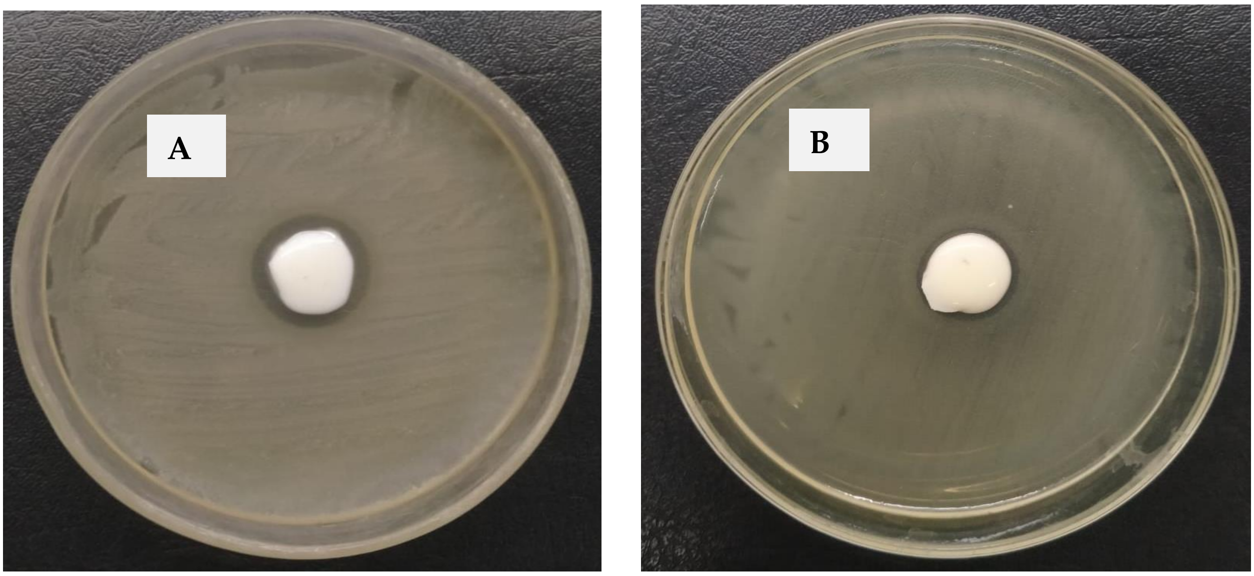

Figure S4. (A and B) is activity of nanoemulgel against *Pseudomonas aeruginosa* and *Bacillus chungangensis* respectively.

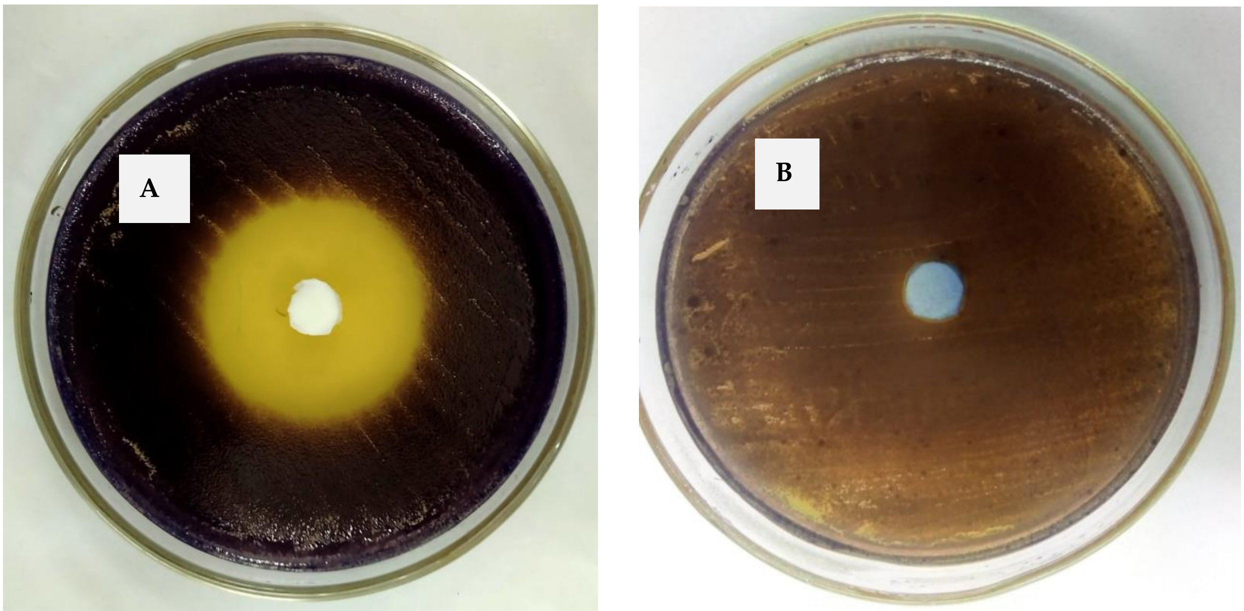

Figure S5. (A and B) Antiquorum sensing activity of essential oil loaded and un loaded nanoemulgel respectively against *Chromobacterium voilaceum*.
